# Research capacity of global health institutions in China: a gap analysis focusing on their collaboration with other low-income and middle-income countries

**DOI:** 10.1136/bmjgh-2021-005607

**Published:** 2021-07-15

**Authors:** Xiaoxiao Kwete, Kun Tang, Feng Cheng, Yingyao Chen, Yuan-Tao Hao, Zongfu Mao, Ran Ren, Yunping Wang, Youfa Wang, Chenkai Wu, Dong (Roman) Xu, Ying Zhao, Xiao-Nong Zhou, Yuning Liu, Ruoyu Yin, Xiaohui Liang, Chun Hao, Yayi Guan, Yangmu Huang, Man Tat Alexander Ng, Peilong Liu, Yemane Berhane, Wafaie Fawzi, Zhijie Zheng

**Affiliations:** 1 Global Health and Population, Harvard University T H Chan School of Public Health, Boston, Massachusetts, USA; 2 Global Health Research and Consulting, Yaozhi, Yangzhou, China; 3 Vanke School of Public Health, Tsinghua University, Beijing, China; 4 Department of Hospital Management, Fudan University, Shanghai, China; 5 School of Public Health, Sun Yat-sen University, Guangzhou, China; 6 Sun Yat-sen Global Health Institute, Sun Yat-sen University Institute of State Governance, Guangzhou, Guangdong, China; 7 School of Health Sciences, Wuhan University, Wuhan, China; 8 Global Health Research Center, Dalian Medical University, Dalian, China; 9 China National Health Development Research Center, Beijing, China; 10 Global Health Institute and School of Public Health, Xi'an Jiao Tong University, Xi'an, China; 11 Global Health Research Center, Duke Kunshan University, Kunshan, China; 12 SMU Institute for Global Health and School of Health Management, Southern Medical University, Guangzhou, Guangdong, China; 13 Fudan University School of Nursing, Shanghai, China; 14 National Institute of Parasitic Diseases, China CDC, Shanghai, China; 15 School of Global Health, Shanghai Jiao Tong University School of Medicine & Chinese Center for Tropical Diseases Research, Shanghai, China; 16 Harvard University T H Chan School of Public Health, Boston, Massachusetts, USA; 17 Department of Public Health and Primary Care, University of Cambridge, Cambridge, UK; 18 Department of Global Health, School of Public Health, Peking University, Beijing, China; 19 Institute for Global Health and Development, Peking University, Beijing, China; 20 Tencent Healthcare, Tencent, Shenzhen, Guangdong, China; 21 Addis Continental Institute of Public Health, Addis Ababa, Ethiopia

**Keywords:** health policy, public health, review, qualitative study

## Abstract

**Introduction:**

This paper presented qualitative and quantitative data collected on the research capacity of global health institutions in China and aimed to provide a landscaping review of the development of global health as a new discipline in the largest emerging economy of the world.

**Methods:**

Mixed methods were used and they included a bibliometric analysis, a standardised survey and indepth interviews with top officials of 11 selected global health research and educational institutions in mainland China.

**Results:**

The bibliometric analysis revealed that each institution had its own focus areas, some with a balanced focus among chronic illness, infectious disease and health systems, while others only focused on one of these areas. Interviews of key staff from each institution showed common themes: recognition that the current research capacity in global health is relatively weak, optimism towards the future, as well as an emphasis on mutual beneficial networking with other countries. Specific obstacles raised and the solutions applied by each institution were listed and discussed.

**Conclusion:**

Global health institutions in China are going through a transition from learning and following established protocols to taking a more leading role in setting up China’s own footprint in this area. Gaps still remain, both in comparison with international institutions, as well as between the leading Chinese institutions and those that have just started. More investment needs to be made, from both public and private domains, to improve the overall capacity as well as the mutual learning and communication within the academic community in China.

**Key questions**

**What is already known?**

- Most global health institutions in China were established after 2000 and are growing fast in both scales and scope of work while still facing many challenges.

**What are the new findings?**

- Bibliometric analysis and standardised survey included in this study provided detailed quantifiable measurements of the research capacity of these institutions and respective strengths and weaknesses.
- Enhancing collaboration with other low-income and middle-income countries was a major part of their development strategy.

**What do the new findings imply?**

- Research capacity of global health institutions in China can be improved to a large extent by increasing collaboration with their international partners as well as domestic ones.
- Trilateral partnerships can play an important role in the development of global health in China.

## Introduction

The current COVID-19 outbreak has brought the world’s attention to global health institutions in China, whose efforts on research, public education and policy advocacy have a great impact in shaping how China can work with the world in tackling the biggest threat to face human society today.^1^ Universities in China quickly joined the fight against COVID-19, first by ensuring the safety and continuity of education programmes of students at schools, and later by taking on their social responsibility and contributing via biomedical research, public health interventions, policy making support and public education programmes.^2–4^ On 4 April 2020, Harvard TH Chan School and Wuhan University held a joint seminar online, in which 13 experts on respiratory diseases, virology, epidemiology and health systems from both the USA and China shared their experiences on COVID-19 response.^5^ Other universities have also organised similar events, engaging global health experts from China and other high-income countries (HICs) in strengthening international dialogue and collaboration towards a united global response to this pandemic.^6^ As the numbers of COVID-19 cases are now climbing up in Africa, South Asia and Latin America, it is critical to also look at how global health institutions in China have been conducting research and projects working with other low-income and middle-income countries (LMICs) and how this could affect their ability to take on more global health responsibility as one of the two leading economies in the world.

Since the severe acute respiratory syndrome (SARS) outbreak in 2003, the Chinese government has become more active in participating in global health governance, working with various international organisations on different health issues.^7^ This growing global awareness trajectory goes beyond the health sector as China seeks to establish a more prominent position on the global stage. In 2016, Chinese President Xi Jinping announced ‘Healthy China 2030’, an ambitious agenda to promote health across China that also emphasised innovative models for strengthening South–South cooperation, including ‘The Belt and Road Initiative’ and implementation of the China-Africa Public Health cooperation plan. Africa makes a special mark in the history of China’s global health diplomacy as the earliest health outreach project; Chinese medical teams organised by the Chinese government to work abroad started in 1963 when China dispatched its first medical team to Algeria in North Africa.

Global health is still a relatively new discipline in China. China’s universities started to set up global health centres/institutions in 2007, with Peking University being the first to establish a global health research centre.^8^ By 2014, a few additional universities have set up their global health programmes/departments/institutions/centres: Fudan, Sun Yat-sen, Central South (Xiangya), Wuhan, Kunming, Duke (Kunshan) and Peking Union Medical College.^9 10^ More universities followed suit shortly after that. These institutions quickly formed alliances. In December 2013, the China Consortium of Universities for Global Health (CCUGH) was established by 10 founding universities—Central South University, Chinese University of Hong Kong, Duke Kunshan University, Fudan University, Kunming University, Peking University, Peking Union Medical College, Sun Yat-sen University, Wuhan University and Zhejiang University^9^—embarking on an ongoing momentum of global health development in China’s academic institutions.^8^ By the end of 2018, the number of CCUGH members grew to 25. In 2015, the China Global Health Network was established, involving not only academic institutions but also government agencies and actors from the private sector with interest in global health. The following year, the Chinese Preventive Medicine Association set up its Global Health Branch and further consolidated the collective efforts by the government and academia in promoting and enhancing global health research by China.^8^

Although institutions whose primary focus is on global health did not exist in China until this century, they are predated by decades of China’s health collaboration with other international players, for most of which China was on the receiving end of financial and technical support from HICs.^11 12^ The same trend has been taking place in the private sector as well, with China being the top market in LMICs for global pharmaceutical companies to set up their research and development centres.^13^ As a result, even today most of China’s global health collaboration is with HICs.^14 15^

China has produced a growing number of publications on health that have a global impact. Previous scholars have found that China has a higher pharmaceutical research publication among the BRICS countries (Brazil, Russia, India, China and South Africa)^16^ and in Asia.^17^ Similar studies also recognised China’s leading role among the BRICS countries in research and publication on HIV,^18^ tuberculosis^19^ and other neglected tropical diseases.^20^ Recently, there is also a growing literature on the Belt and Road Initiative,^21^ with a focus on infectious disease surveillance since China’s active involvement in 2014’s Ebola outbreak in West Africa.^22^ However, other scholars think that, although China has been expanding its engagement and influence in global institutions, it is quite limited in contributing to global public goods.^23^ Others discussed that articles published by Chinese scholars are more likely to cite other articles published within China, which suggests a high level of insularism.^19 24^

In general, evidence on the research capacity of Chinese global health institutions is limited. In a recent paper, Xu *et al*^10^ noted barriers that Chinese global health institutions face in their work, including lack of and restrictions on funding, limited human resource capacity, and lack of experience in other LMICs. This article aims to build on that effort and makes a unique contribution by collecting and analysing first-hand quantitative and qualitative data related to research capacity of global health institutions in China, and use the findings to depict a more detailed landscape of the current academic environment in China for the future development of global health.

## Methods

This study consists of three components: a bibliometric analysis of global health publications, a standardised survey of institutional indicators and qualitative indepth interviews of key informants. The bibliometric analysis was conducted using the names of 10 universities/organisations, while the survey and interviews were conducted on the specific departments/institutes of global health at these institutions (see online supplemental table 1 for the list).

10.1136/bmjgh-2021-005607.supp1 Supplementary data

The definition of global health has been evolving over the past decades, with scholars in this field focusing on multidisciplinary collaboration that bridges different subject areas such as epidemiology, biostatistics and social science, with a focus on interdependence and reducing health inequity globally.^25^ While we recognise that global health research includes a wide spectrum of studies on health issues across the globe, we applied a working definition of global health research based on Koplan *et al*’s description,^25^ but with two executable criteria: (1) the research is conducted in or on LMICs, with at least one LMIC other than China involved, using the most recent classification of country income groups by the World Bank^26^; and (2) the research is in at least one the following areas: (1) maternal and child health (MCH), (2) malaria and other infectious diseases (ID), (3) disease surveillance, (4) non-communicable diseases (NCDs), (5) health system strengthening (HSS), (6) environmental health (EH), and (7) international aid, development and global governance (AD), as was agreed by the author group prior to this study.

### Bibliometric analysis

The names of the 10 universities/organisations were used in the bibliometric analysis rather than the specific global health institutions/departments because (1) most of the faculty conducting global health research have a primary affiliation with another general department, in most cases at a school of public health or school of medicine. Only searching for publications by these global health institutions would miss many relevant papers, and (2) publications on global health by other scholars in the same university are also an indicator of the global health research capacity of that institution. The search was conducted in three English databases and one Chinese database: PubMed, EMBASE, Web of Science (Thomson) and WanFang (see online supplemental table 2 for the search terms used and online supplemental table 3 for the names of institutions used in the search).

Titles and abstracts were reviewed independently to select articles on global health topics in the above noted seven areas. The addresses/affiliations of all selected articles were reviewed to make sure those articles are published by those institutions and related to global health.

Data extraction was carried out by two researchers (XK and YL) independently using a predetermined data extraction table with the following categories: study identifiers (author, year of publication, title) and study characteristics (institution affiliations, language, country/regions, thematic areas). One article can be categorised into one or more of the seven focused areas as used in the definition of global health. Five regions of LMICs were used: (1) Africa, (2) Asia and Pacific, (3) East Europe, (4) Latin America and (5) Middle East. An article will be categorised as (6) global if the article involved more than one of the five regions of LMICs. Differences between the two researchers were resolved with group discussion.

### Standardised survey

The survey was designed to understand the workforce, programmes, publications and collaborations at global health institutes in China. To map the research capacity of each institution, we developed a questionnaire that contains structural indicators, process indicators and outcome indicators. Structural indicators included information about researchers and their degrees, backgrounds and grants. Process indicators investigated the training and exchange programmes offered, as well as the symposia organised by these institutions. Outcome indicators measured the publications, conference presentations, postdoctoral training and foreign-based projects of each institution. Institutions that agreed to participate within the timeline provided were included in both the survey and the following indepth interviews. The head of that institution was asked to designate an appropriate person with the knowledge to fill out the standardised survey. Data were summarised by category and by institutions. Descriptive data such as names of sponsoring institutions were categorised and presented in graphs.

### Key informant interviews

Indepth interviews were designed to gather information about the barriers faced, experiences gained, support needed and academic expectations of global health institutions in China. Invitation letters were sent to all 11 institutions between February and July 2018. All of them replied and accepted the interview. The positions of each person interviewed were reviewed to make sure they have enough knowledge about the institution to answer the questions. Each interview lasted 60–90 min, where interviewees answered multiple questions on their understanding of global health research, institutional objectives, barriers, solutions applied so far and external support required (see online supplemental table 5 for the interview guides used). XK and KT conducted the interviews in Chinese, and full transcripts were translated to English and sent back to the interviewees for their review and approval. Results from the interviews were summarised by question and are merged or compared for further analysis. Conventional qualitative content analysis method was used to analyse the data extracted from selected articles.^27^ Original texts were used to avoid biases when recording them. Keywords were extracted as they emerged from the text, with no predetermined framework, and were then compared with each other to collate into a few categories. Themes were summarised from those categories as agreed by the research group.

## Results

### Bibliometric analysis of global health publications

The bibliometric search yielded a total of 21 501 articles from 10 institutions after duplicates were removed, published before 21 August 2018. After the title, abstract and full-text review, a total of 1085 articles were included in the final analysis (see online supplemental table 4 for details).

**Figure 1:**
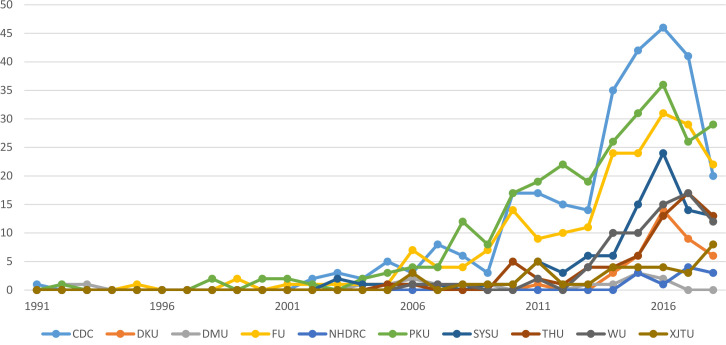
Number of publications on global health from 10 institutions in China. CDC, China Center for Disease Control and Prevention; DKU, Duke Kunshan University; DMU, Dalian Medical University; FU, Fudan University; NHDRC, National Health Development Research Center; PKU, Peking University; SYSU, Sun Yat-sen University; THU, Tsinghua University; WU, Wuhan University; XJTU, Xi’an Jiao Tong University.

The total number of publications on global health revealed a clear ‘first tier’ global health institution in China: China Center for Disease Control and Prevention (CDC), Peking University and Fudan University (see figure 1). This finding was also confirmed in the key informants interviewed in later sections. The trends showed that the ‘first tier’ group began to emerge from the rest of global health institutions in China in 2006 and 2007 and the gap widened in 2010 and then in 2014 (see figure 1).

Institution-specific analysis showed that each global health institution had its own distinctive thematic areas. For example, global health research at the China CDC had a clear focus on ID, which took up more than 68% of their total publication records. They also had invested quite a few resources on NCDs, with about 20% of publications. Duke Kunshan University had a focus on NCDs, with more than 60% of publications in this area, followed by 23% of publications on HSS and 18% on ID. Duke Kunshan University was the only institution included in this study with more than 50% of publications on NCDs. This positioned Duke Kunshan University to be a key collaborator on NCD-related projects in Africa and other LMICs. Dalian Medical University did not yield a big number of publications, but showed a heavy focus on HSS (60%) and a good balance among four other subjects: MCH (13%), ID (13%), NCDs (7%) and AD (13%). Fudan University had almost equal number of publications going to ID (26%) and NCDs (24%), followed by HSS (17%), MCH (16%), AD (15%) and EH (7%). Not surprisingly, more than half of the National Health Development Research Center’s (NHDRC) publications were on HSS in other LMICs, and another 36% addressed AD. Note that NHDRC is a government-affiliated institution, so a large proportion of their research were not turned into publications, but rather served as internal reference and policy briefs for government departments. Peking University’s leading global health area in LMICs was NCDs (37%), followed by MCH (20%), HSS (19%), ID (14%) and AD (10%). A small percentage (3%) went to EH. Sun Yat-sen University had a clear emphasis on disease-specific research, with ID and NCDs taking up 41% and 35%, respectively; it was the only institution among those selected in this study where both ID and NCDs took up more than 30% of the total articles. Tsinghua University was unique among others in that it had a leading position on EH, especially in relation to global warming and global reconciliation mechanisms to combat global warming, and generated many publications on global governance. As a result, both EH and AD took up 27% of the total articles, followed by ID at 24% and HSS at 20%. Tsinghua University’s advantage in science and technology put them in a unique position to continue leading the area of EH and other global health efforts in fighting against the negative health impacts brought by climate change. Wuhan University had a similar subject breakdown compared with Peking University: NCDs took up the leading position (36%) and ID took up a smaller proportion (18%), while HSS and MCH each took up around 20%. More global health articles published by teams at Xi’an Jiao Tong University were on ID (39%) and MCH (31%) than NCDs (22%); HSS took up another 14% (see table 1 for more details).

**Table 1:** Number of global health publications from 10 universities/organisations, by thematic and geographical areas

	CDC		DKU		DMU		FU		NHDRC		PKU		SYSU		THU		WU		XJTU	
	n	%	n	%	n	%	n	%	n	%	n	%	n	%	n	%	n	%	n	%
MCH	20	7	2	5	2	13	32	16	1	9	52	20	7	7	3	5	16	22	11	31
ID	189	68	7	18	2	13	53	26	0	0	38	14	39	41	16	24	13	18	14	39
DS	12	4	1	3	0	0	1	0	0	0	2	1	1	1	1	2	0	0	2	6
NCDs	56	20	25	64	1	7	49	24	1	9	98	37	33	35	5	8	26	36	8	22
HSS	17	6	9	23	9	60	35	17	6	55	50	19	6	*6*	13	20	17	23	5	14
EH	12	4	2	5	0	0	14	7	0	0	8	3	12	13	18	27	2	3	0	0
AD	0	0	0	0	2	13	31	15	4	36	27	10	10	11	18	27	10	14	1	3
Africa	49	18	2	5	3	20	25	12	0	0	24	9	15	16	10	15	3	4	7	19
Asia and the Pacific	84	30	9	23	3	20	55	27	2	18	70	26	38	40	19	29	18	25	7	19
Latin America	1	0	2	5	0	0	4	2	0	0	6	2	2	2	2	3	0	0	1	3
Middle East	1	0	0	0	0	0	1	0	0	0	0	0	0	0	0	0	4	5	0	0
East Europe	0	0	1	3	0	0	1	0	0	0	0	0	0	0	0	0	0	0	0	0
Global	145	52	25	64	9	60	118	58	9	82	166	62	39	41	35	53	48	66	21	58
Total	280		39		15		204		11		266		94		66		73		36	

Institutional breakdown: 
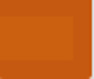
 >30%; 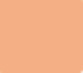 10%–30%; 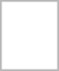 <10%.

AD, aid, development and global governance; CDC, China Center for Disease Control and Prevention; DKU, Duke Kunshan University; DMU, Dalian Medical University; DS, disease surveillance; EH, environmental health; FU, Fudan University; HSS, health system strengthening; ID, infectious diseases; MCH, maternal and children’s health and nutrition; NCDs, non-communicable diseases; NHDRC, National Health Development Research Center; PKU, Peking University; SYSU, Sun Yat-sen University; THU, Tsinghua University; WU, Wuhan University; XJTU, Xi’an Jiao Tong University.

Geographically speaking, there was much less variance among these global health institutions. Global health research that involved more than two low-income and middle-income continents took the most part of all publications. It is also worth noting that in most of these cross-continental collaboration research projects, such as the Global Burden of Diseases Studies, SAGE (Study on Global Ageing and Adult Health), International Union Against Tuberculosis and Lung Disease, Global Project on Anti-Tuberculosis Drug Resistance Surveillance, and WHO-recognised national influenza centres, scholars from Chinese institutions played more supporting roles than leading roles. Very few of those publications listed scholars from Chinese institutions as first or corresponding authors.

For articles where only one low-income and middle-income continent was involved, Asia ranked the highest in terms of articles, followed by Africa, with very minimal publications for other continents: Latin America, Middle East and East Europe. This pattern was almost identical across all institutions, with very slight differences.

### Standardised survey

Nine out of eleven standardised surveys were completed, and two of nine only had partial data due to time availability issues of faculty members. Only data that were later validated by the study group were selected for presentation. For example, self-reported number of global health publications was dropped from the table, since each institution might have used a different definition of global health when counting publications.

Although ‘global health institutes or departments’ were established, the number of full-time professors at all surveyed institutions was limited (see tables 2–3). Not all schools offered courses on global health for undergraduates and graduates. Even within schools that had courses, most of them were introductory courses, revealing limited capacity in global health teaching.

**Table 2:** Research and education resources from selected global health institutions in China

	Faculty (n)	By degree								
		M	D	F	G (2006–2017)	C-SF	C-S	T-D	T-I	IRB
CDC-NIPD	12	0	12	0	24	NA	NA	8	6	NA
DKU	10	0	10	5	22	9	NA	2	0	NA
DMU	5	0	0	0	1	8	0	0	0	0
FDU-N	11	5	6	0	2	0	0	0	0	1
FDU-PH	4	0	1	0	7	0	0	23	0	1
PKU	9	1	8	0	15	5	4	3	0	1
SYSU	13	0	14	2	3	2	NA	1	0	1
XJTU	9	1	8	0	17	5	0	0	0	0
THU	2	0	2	0	4	8	0	20	0	0

C-SF: number of curriculum on global health offered to undergraduate and postgraduate students during spring/fall semester; C-S: number of curriculum on global health offered to undergraduate and postgraduate students during summer semester; T-D: number of training on global health offered to domestic executives or officials; T-I: number of training on global health offered to international executives or officials; IRB: is IRB set up to review relevant studies? (1=yes, 0=no).

CDC-NIPD, China Center for Disease Control and Prevention, National Institute of Parasitic Diseases; D, doctor’s degree or above; DKU, Duke Kunshan University; DMU, Dalian Medical University; F, foreign researchers; FDU-N, Fudan University School of Nursing; FDU-PH, Fudan University School of Public Health; G, grants received on global health; IRB, Institutional Review Board; M, master's or above; NA, not applicable; PKU, Peking University; SYSU, Sun Yat-sen University; THU, Tsinghua University; XJTU, Xi’an Jiao Tong University.

**Table 3:** Research and education outcomes from selected global health institutions in China

	SE	S	P	PG: 2006–2017	PD: 2006–2017	African partners	DC
CDC-NIPD	4	13	NA	NA	18	7	1
DKU	NA	5	NA	NA	8	4	1
DMU	1	5	6	3	1	0	1
FDU-N	0	1	1	0	0	0	0
FDU-PH	0	9	0	0	0	0	0
PKU	1	2	0	1	0	3	0
SYSU	7	5	3	11	0	1	1
XJTU	2	2	6	78	7	0	0
THU	1	1	0	77	0	2	0

SE: number of scholar exchange programmes with other low-income and middle-income countries; S: number of symposia organised in global health; P: number of conference presentations in global health; PG: number of postgraduate students trained in global health; PD: number of postdoctoral fellows trained in global health; DC: domestic collaboration: are you collaborating with another global health institute in China on global health projects? (1=yes, 0=no).

CDC-NIPD, China Center for Disease Control and Prevention, National Institute of Parasitic Diseases; DKU, Duke Kunshan University; DMU, Dalian Medical University; FD-PH, Fudan University School of Public Health; FU-N, Fudan University School of Nursing; PKU, Peking University; SYSU, Sun Yat-sen University; THU, Tsinghua University; XJTU, Xi’an Jiao Tong University.

**Figure 2:**
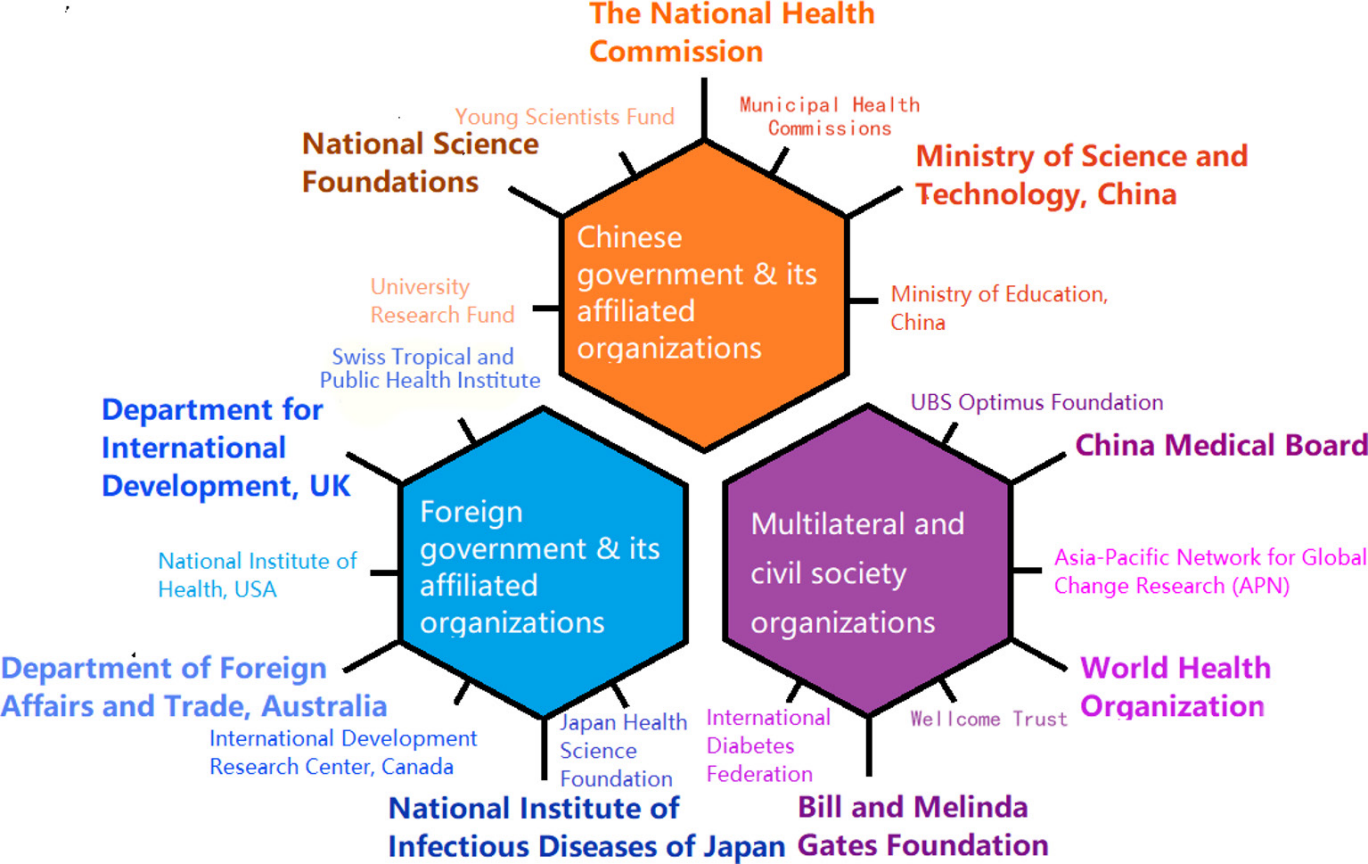
Sources of funding for global health institutions in China.

In general, the number of grants received for global health has been increasing for all nine institutions surveyed. Although the amount of grants fluctuated over the years, the overall trend indicated an upward trajectory. Besides Peking University and Fudan University, Xi’an University and Sun Yat-sen University also received comparable amounts of funding for global health. In terms of sources of funding, international sponsors played an indispensable role; all surveyed institutions had at least one sponsor outside of China. The biggest sponsor was the UK government through the Department For International Development. The funders of those grants generally fell into three categories: (1) Chinese government and its affiliated organisations, (2) foreign government and their affiliated organisations, and (3) civil society organisations. Each category included a variety of players, some of which have supported more global health programmes in China than others (see figure 2).

Some schools prioritised organising short-term training courses or holding symposia to teaching curriculum to full-time students (eg, Fudan offered 23 short-term training courses and 0 curriculum for students during the surveyed time), which showed the different development strategies in the surveyed institutions. Others such as Dalian Medical University organized periodic extra-curricular seminars on global health that was not included this table. All nine institutions had international partners in global health, including multilateral organisations such as WHO. A few institutions such as Peking University and Sun Yat-sen University listed partnering institutions in Africa. However, the number of their African partners was dwarfed by that of global partners in other regions (see table 3 for more details).

### Key informant interviews

When asked about their understanding of global health research, representatives from all 11 institutions mentioned key features such as ‘cross border’, ‘collaboration’ and ‘multi-disciplinary’. In addition, a few key common features of the China-Africa health collaboration were recognised by the majority of interviewees, including (1) a historical friendship that goes beyond the health sector, (2) long history of China-Africa health collaborations, (3) current high level of political will, (4) featuring the application of China’s success and experience in health development to Africa together with international organisations, and (5) a foundation of mutual benefits.

Besides Africa, there hasn’t been another continent in the world, to which China has systematically and consecutively sent medical teams.

Before, we used to stress the word “cooperation”, which means each side comes with their own interests and make the transaction; now we stress the word “collaboration”, which emphasize on shared value and shared goals. China-Africa Health Collaboration should be based on this spirit.

Among these key common features, many interviewees emphasised the unique position of Africa in their global health outreach strategies, as illustrated by the quotes above.

Most institutions had comprehensive goals for their global health capacity, including promoting health, research/knowledge generation, education and training, technical support and consultation, and policy support for the Chinese government, while some institutions had priorities of one or two over the others. For example, the Global Health Institute at Wuhan University indicated a strategic focus on human resource training (see online supplemental table 6 for more details).

Barriers faced collectively by all institutions to conducting global health research, especially in relation to LMICs and Africa specifically, were identified and described in detail below. Experiences shared to overcome each barrier are listed as well. No institution claimed that they have overcome all barriers to an extent they are satisfied with. Rather, many interviewed said that, for some of the barriers, they have not come up with any solutions at all (see box 1 for the direct quotes from the indepth interviews). The barriers noted included the following:

1. Lack of policy and political support: No clear guidance on global health diplomacy for China, so researchers in this field felt that they did not have a clear understanding, especially for institutions directly affiliated with the Chinese government. In addition, China’s National Health Commission had relatively weaker political power within the government system, which made it difficult to convince and mobilise other non-health sectors to support decision making and project implementation in health. It was noted that global health research and projects involved cross-border activities, and almost all Chinese universities, with very few exceptions, were public universities, which meant that international trips had to be approved by the government before taking place.
2. Lack of partners/networks in LMICs: Many institutions stressed the importance of having a close and day-to-day partner for their research project in LMICs. Without a local partner, it was difficult to conduct population-level interventions that required a much deeper understanding of the community. Also, they noted that it was crucial to develop appropriate technologies with a local partner, who had detailed knowledge of the context, including applicable technologies, regulations of medicine and medical devices, functions of delivery platforms, etc. Due to lack of understanding of other countries, it was difficult for Chinese global health institutions to identify good partners to start with. Even when they did, their partners usually suffered from lack of resources, which highly restricted their capability in the collaboration.
3. Lack of knowledge on local context, language and culture: Even with a good local partner, one needed to have a decent understanding of the local context and culture in order to design and implement global health research. Local context included but was not limited to how people interpret medical terms, how each level of healthcare system functioned, how medical products and practices were regulated, and the specific epidemiological and demographic profile of the local population. In addition, China had a well-functioning data collection system following the SARS outbreak on IDs and beyond, which most other LMICs did not have. So it was difficult to expect things would happen as they did domestically. Most interviewees compared their experience in other Asian countries with that in Africa and emphasised the higher sense of distance between the cultures of China and Africa, compared with that in other Asian countries. In addition, not having adequate knowledge of the local legal, political and academic structures also exacerbated the barriers to identifying and establishing a good partner/network in those LMICs. It also generated safety concerns for researchers conducting research on the ground.
4. Lack of experienced faculty and trained students/researchers: Many faculties in those global health institutions had education and/or training background in HICs, but not in other LMICs. Many of them did not have any experience conducting research in another LMIC. Not many universities in China provided systematic training on global health for undergraduate or postgraduate students.
5. Funding limitations: Interviewees highlighted limited funding sources for global health research in China, with the exception of the UK-China Global Health Support Program, to which Peking University and Fudan University were the two main recipient institutions. The current South–South Collaboration Fund did not have funding that targeted global health, nor were there other major national funds, such as the National Natural Science Foundation of China, the National Social Science Fund of China, Major Technology Programme Fund, etc. Also, even if these institutions were given funding to do global health research, these funds had limitations on overseas expenses, which largely restricted research planning and implementation. Currency exchange was also a challenge, since renminbi to US dollar exchange was highly regulated in China and funding in renminbi can only be used abroad after being converted to US dollars.
6. Inappropriate credit system for young faculty: Many interviewees mentioned that the current credit system in Chinese universities was not favourable to faculty doing global health research in another LMIC. For example, if a young faculty spent about a year in an institution in an HIC, that experience would be counted as ‘advanced training’, which added to the profile for future promotion considerations. However, if he/she spent that year in another LMIC, it would not count.

Box 1 **Quotes from indepth interviews: barriers to global health research**

- “China has years of experience of sending medical teams to Africa, and on how Chinese people working abroad. So, you have policy guidance on how to arrange people and projects. But for public health, we don’t have any policy guidance. When we explore those collaborations, we need to bring our experiences back to policy makers in China, to help them make relevant policies in the future.”
- “We want to find suitable partner and to establish cooperative relationship with some African countries. But now we haven’t. We need more data and information for African countries.”
- “Also, the culture of Africa is difference from ours. When we visit South East Asian countries, we feel we share a lot of things with them, so we can easily understand what and how people think. But it’s different in Africa. And Africa is not just one country but a variety of different countries with different background.”
- “Our team is almost 100% educated in Europe and North America. They are very familiar with developed countries, but only macro-level understanding of societies, economies and health issues in developing countries. They lack the experience of doing on-the-ground work in those settings.”
- “There is little funding opportunity from within China to fund research abroad. Very rarely do global health institutions from China conduct research abroad. So far, DFID is the biggest sponsor in this area, and the funds mainly went to PKU and Fudan. But still, the DFID funds were essentially for service delivery, not research-oriented, which is very rare, and not available within the National Science Funds of China.”
- “In the current evaluation system in universities, the criteria for promotion are mostly number of publications, teaching workload, number of funds etc. And that poses a great challenge to global health research. We need to go to another country and truly help local people, to be able to do global health research, not to remotely control from China. They’d better stay there for one or two years. But, with the current evaluation and promotion system, it’s difficult. If they choose to go to a developed country for one or two years, it will be considered advanced training, and it adds points to his or her evaluation, but if they choose to go to Africa, it will not only add points but deduct points, because it will affects his or her teaching work and number of publications. We face barriers in encouraging young faculty to do global health research.”

Different solutions that tackle each of the barriers presented above are listed in table 4; they were used by the interviewed institutions to overcome barriers, although these were noted to be inadequate to resolve the concerns.

**Table 4:** List of barriers and solutions for global health research at Chinese institutions

Barriers	Solutions taken by global health institutions in China
Lack of policy and political support	Visit the local Chinese embassy before you start your project.
	Mobilise other ministries within the Chinese government by inviting them to meetings and explaining how global health research can help the development of their respective sectors.
	Use one’s own network within the political system to make things happen, although the impact might be small.
	Have higher level agreement signed (at ministerial level) before you start your project.
Lack of partners in LMICs	Start from simple, descriptive and small-scale projects and gradually move on to more complicated research.
	Send team from China to work together with local partners.
	Collaborate with partners from HICs.
	Ask bilateral agencies from HICs or multilateral agencies that have experience working in those LMICs to recommend partners.
	Build strong relationship with our existing partners through mutual respect.
Lack of knowledge on local context, language and culture	Make use of overseas students in the campus.
	Recruit faculty member from other LMICs.
	Get trained by other schools or department with knowledge of the local community, such as social scientists and anthropologists of African culture and religion.
	Collaborate with partners from HICs.
	Make more friends through collaborations.
	Get trained by the local Chinese embassy before your start your project.
Lack of experienced faculty	Recruit faculty from other LMICs.
	Recruit faculty with experiences in LMICs.
	Start from simple, descriptive and small-scale projects and gradually move on to more complicated research.
	Collaborate with partners from HICs.
	Organise lectures, symposia and conferences to attract more young students and scholars joining global health.
	Provide internal training to staff members.
Funding limitations	Fund-raise from within the university.
	Fund-raise from both public and private sectors.
	Work together with the school’s management department to think of creative ways to use the fund.
	Collaborate with partners from HICs, such as the UK, USA and the Netherlands.
Inappropriate credit system for young faculty	Build a strong team with strong team spirit.
	Support those faculty with their publications.

HICs, high-income countries; LMICs, low-income and middle-income countries.

Each institute also listed ways external support could be provided to strengthen their capacity in global health research. Interviewees mentioned different stakeholders, which we summarise in table 5.

**Table 5:** External support required to strengthen global health research capacity

Stakeholders	Support
Chinese government	To provide more funding in global health.
	To provide more logistical support for projects running abroad.
	To more closely integrate health into other foreign aid programmes of the Chinese government.
	To have clear national strategies and policy guidance for China’s aid projects.
International/domestic foundations and organisations	To provide more funding in global health.
	To build capacity to assist research institutions in implementing global health projects in other LMICs (for Chinese organisations).
	To have more staff exchange programmes with the Chinese government and other organisations.
	To recognise and promote the efforts and progress made by China’s global health institutions.
International academic institutions	To organise training on: How to apply for global health funds, how to collaborate with local teams, etc. Intercommunication skills and cultural issues. Basic global health concepts. Epidemiological and health systems of LMICs.
	To organise short-term similar/workshops.
	To have exchange scholar programmes.
	To collaborate on global health projects: apply for funds together, design the study together and write report/articles together.
	To organise trilateral partners, sharing resources, networks, channels and experiences with China, while also learning from how Chinese conduct global health research/programmes.
	To recognise and promote the efforts and progress made by China’s global health institutions.
	To form closer and long-term research partners.

LMICs, low-income and middle-income countries.

Common features were also identified about their expectations of research capacity in global health in the next 5–10 years. In addition to a strong will to improve the overall research capacity, most interviewees were very optimistic about the development of overall research capacity that would enable them to grow out of the current ‘infant phase’. Many emphasised that they expected to grow their impact and establish closer ties with other LMICs, especially Africa, and expand on existing networks or establishing more cross-region academic networks within the next decade (see online supplemental table 7 for details).

## Discussion

This study confirmed previous research that global health institutions in China are in their early development and are facing many barriers.^28^ Our bibliometric analysis showed that, before 2004, none of those institutions had a sizeable publication record on global health, which corresponds to previous scholars’ observation on how Severe Acute Respiratory Syndrome (SARS) had played an important role in sparking China’s engagement in global health.^7^ The number of full-time professors at all surveyed institutions was limited, which shows both the need to prioritise global health in universities and the deficiency of global health workforce. Our study also confirmed the huge variance in development among these domestic global health players, as has been published by other scholars before in other health-related fields.^28^ Both the different focus and an internal gap suggest the potential of fostering collective growth by learning from each other. Institutions not included in this study could also use this report as a reference to position themselves.

To this date, the Natural Science Foundation of China has not released global health-focused research funds despite strong and long-standing advocacy for that from universities and research institutions. Results from our standardised survey showed that most of the funding for global health comes from bilateral agencies or private international foundations, rather than domestic agencies, public or private, which is corroborated by findings from earlier research.^10^ As the Chinese government is investing more in its global strategy, more resources need to be appropriated to the health sector to provide a balance to the economic-driven approach that has attracted heavy criticism. In addition to more diverse funding sources, the results from this article also indicate a growing need for the Chinese government to set up clearer guidelines on global health strategies and establish proper non-capital incentives such as career advancement for researchers and educators working on global health to expand their impact to other LMICs.

Global health institutions in China are at different stages of development, and this study showed that there are early signs of strength area for each institution: some have a balanced investment in different thematic areas, while others have clear focus of research and practice in one or two of them. For example, NHDRC’s strength is on HSS, China CDC on ID and Tsinghua University on EH. Peking University and Fudan University both have a balanced record across different subject areas, with Peking University having a slight edge in NCDs and Fudan University in ID. This information will help guide future policy making and strategising, both for individual institutions as well as for the country. We highly recommend strategic partnering among domestic global health institutions which could immediately expand the portfolio of what kind of research can be conducted and the quality of those research or outreach global health projects to other LMICs. Some of the recent publications already showed this trend.^29^

Most of the publications yielded in this study involved multiple continents where the Chinese institutions serve as one of the partners for data collection. More detailed analysis needs to be done on the database to get more insights on what roles Chinese institutions played in these cross-continental global health collaborations. These publications were followed by some publications with partners in Asia, a smaller number of publications in Africa, and a minimal amount of publications in other areas such as Latin America and the Middle East. We thus suggest that global health institutions in China make use of the experiences they have accumulated in HICs and other Asian countries when they seek expansion of their global partners to Africa and the rest of the world. As shown by the results from the interviews, this is also a strategy that global health institutions in China are very willing and are actively seeking to adopt: to improve their capacity to generate global public goods and to communicate with other LMICs better by strengthening the existing partnerships with and continuing to learn from those institutions in HICs.

In 2015, the Chinese government announced that they are joining hands with the US government in providing technical support to African Union for building an Africa Centres for Disease Control and Prevention.^30^ This example showed the resolve from both China and the USA to be responsible great powers on the global stage that we continue to need today, if not more, in the face of COVID-19, the greatest danger to globalisation human beings have ever encountered. As the tension between the developed world led by USA and China continues to rise in a global economic and public health crisis, and the spirit of competition seems to have triumphed over the spirit of cooperation, global health institutions on multiple sides can play a role to bridge the gap in a trilateral way. We propose that multilateral collaborations could take the form of collaborative research, multichannelled sponsorship, as well as training programmes that rotate across different institutions, as was suggested by multiple participants during the interviews.

Last but not least, our findings showed a shared interest across all global health institutions in China to establish and strengthen research collaborations with partners in Africa in the context of a long history of friendship between the two sides. While the history of a strong health collaboration between China and Africa has been well documented,^31^ recent news of increased tension between Chinese communities in Africa and Africans,^32^ as well as African communities in China and Chinese,^33^ have brought concerns to continuing the traditional friendship in modern days and indicated a fissure that needs to be addressed in order not to hamper future links. On the other hand, as many global health institutes in China are actively seeking collaborators in Africa, this study also provides valuable information to global health institutes in Africa to strategise on which Chinese institutes to collaborate with and in what areas, for mutually beneficial outcomes.

Many established global health organisations are reflecting on the element of supremacy in the practice of global health in modern days, and calls for ‘the decolonization of global health’.^34^ This ongoing movement continues to emphasise that global health is built on the basis of equity and justice, a principle China states it has been following in dealing with other LMICs since the Mao’s era and has reiterated through the promotion of South–South collaborations and the Belt and Road Initiative.^35^ Chinese officials draw attention to China’s own experience as being colonised and as the recipient of multiple global health aid projects in the past century. All partners engaged in global health, including those in China and other HIC settings, need to be cognizant of the legacy of colonisation and work as partners in a way that advances shared learning and form a global health solidarity that is truly based on equal footing of all stakeholders.

This study has a few limitations. First, new global health institutions continue to emerge in China in recent years, including between the completion of our study and the publication of this paper, such as the Global Health Center at China’s CDC, and School of Global Health cosponsored by Shanghai Jiao Tong University School of Medicine and Chinese Center for Tropical Diseases Research. As a result, although we have included the input of key players in global health research in China at the time, whether the information depicted in this paper is exhaustive warrants further investigation in future research. In addition, only data from global health institutions in China were collected and most data were qualitative; thus, the results only showed a limited picture of the ‘gaps’, both within those institutions and with their counterparts outside of China, especially in HICs. For example, if information on funding size was also collected, we would be able to generate ‘funding to impact’ ratio and compare across institutions. Our limited scope of definition of global health also ruled out a large number of publications on China’s domestic health issues or those on the health issues in HICs. We hope this study can pave the foundation for more future research on global health institutions in China, Africa and other countries that would ultimately foster more effective and efficient global health collaborations.

Global health development involves not only academic and research institutions, but also a wide variety of public, private and civil society organisations, many of which are emerging in China in recent years. With the newly established China Agency on International Development and Collaboration, as well as an increasing number of Chinese entities going to Africa or other LMICs, led by or influenced by the Belt and Road Initiative, more research is needed to better understand the landscape in which the plethora of players have huge potential health and social impacts.

Growing opportunities await global health in China, as China seeks to play a more significant role on the international stage, transitioning from mainly a recipient of funding and technical support from other HICs to a global leader and resource provider. Trilateral platforms that use both China’s past collaboration with institutions in HICs as well as the existing bilateral and multilateral networks led by those institutions have a unique place in this landscape. The China-Harvard-Africa Network was established as such an example for knowledge sharing, capacity building and finding solutions to improve health system performance in both China and Africa.^36^ This paper serves towards that purpose as we continue to work on pursuing further understanding of global health institutions in China and Africa to foster stronger partners.

### Patient and public involvement statement

Patients or the public were not involved in the design or conduct of our research, but we plan to involve the public and related stakeholders in the further reporting and dissemination of our research. Full report of our research has been shared with Bill and Melinda Gates Foundation and needs to abide by their respective policies. Key findings from this research will be shared on the website of Harvard TH Chan School of Public Health, and we will actively reach out to institutions to which our research can be beneficial.

## Data Availability

Data from the literature analysis and further de-identified data from the interviews are available upon request. Please email xij029@mail.harvard.edu if you need access to more data used in this article.
