# Supplementary material for: Research capacity of global health institutions in China: a gap analysis focusing on their collaboration with other low-income and middle-income countries
Source: BMJ Glob Health. 2021 Jul 15;6(7):e005607. doi: 10.1136/bmjgh-2021-005607 (PMC8286742; doi:10.1136/bmjgh-2021-005607)
Supplement: Supplementary data [file bmjgh-2021-005607supp001.pdf]

Title

Gap Analysis of The Research Capacity of Global Health Institutions in China; focusing on their collaboration with other low- and middle-income countries

Authors

Xiaoxiao Jiang Kwete,[1,*] Kun Tang,[2] Feng Chen,[2] Yingyao Chen,[3] Zongfu Mao,[4] Ran Ren,[5] Yunping Wang,[6] Youfa Wang,[7] Chenkai Wu,[8] Dong Roman Xu,[9] Ying Zhao,[10] Xiaonong Zhou,[11] Yuning Liu,[12] Ruoyu Yin,[13] Xiaohui Liang,[4] Chun Hao,[9] Yayi Guan,[11] Yangmu Huang,[14] Alex Ng,[15] Peilong Liu,[14] Yemane Berhane,[16] Wafaie Fawzi,[1, †] Zhi-Jie Zheng[14, †]

1. Harvard T.H. Chan School of Public Health, 2. Tsinghua University Research Center of Public Health, 3. Fudan University School of Public Health, 4. Wuhan University School of Public Health, 5. Dalian Medical University, 6. China National Health Development Research Center, 7. Xi’an Jiaotong University Global Health Institute, 8.Global Health Research Center, Duke Kunshan University, 9. Sun Yat-sen University Institute for Global Health. 10 Fudan University School of Nursing, 11. National Institute for Parasitic Diseases, China Center for Disease Control and Prevention, 12. JP Morgan Chase Institute 13. Peking University School of Health Humanities, 14. Peking University School of Public Health, 14. Tencent Healthcare, 16. Addis Continental Institute of Public Health

[*] Correspondence to: Xiaoxiao Jiang Kwete, Harvard T.H. Chan School of Public Health, xiaoxiao@mail.harvard.edu, +1-6174605062

Keywords: China, global health, gap analysis

Supplementary Materials:

Appendix Table 1. list of institutions included in this study

Name of universities/organizations	Name of departments/institutes
China National Health Development Research Center (NHDRC)	N.A
Dalian Medical University (DMU)	Institute for Global Health
Duke Kunshan University/Duke University (DKU)	Global Health Research Center
Sun Yat-sen University (SYS)	Institute for Global Health
Tsinghua University (THU)	Research Center of Public Health
Fudan University (FU)	School of Public Health
Fudan University (FU)	School of Nursing
Peking University (PKU)	School of Public Health
Wuhan University (WU)	Global Health Institute
China CDC (CDC)	National Institute for Parasitic Diseases
Xi'an Jiaotong University (XJTU)	Global Health Institute

Appendix Table 2. Search terms used for the literature review on bibliometric analysis of global health publications by selected Chinese institutions

Database	Search terms
Pubmed	((“global health”[tiab] OR “global governance”[tiab] OR “global diplomacy”[tiab]) OR ((Health [tiab] OR Maternal [tiab] OR child [tiab] OR children [tiab] OR Malaria OR Infectious [tiab] OR “disease surveillance” [tiab] OR “Health policy”[tiab] OR “health system” [tiab] OR “health systems”[tiab] OR “chronic illnesses” [tiab] OR “non-communicable” [tiab] OR NCD [tiab] OR “Environmental health” [tiab] OR “Climate change” [tiab] OR “International development” [tiab] OR aid [tiab]) AND (“Low income” [tiab] OR low-income [tiab] OR middle-income[tiab] OR “middle income” [tiab] OR “resource constraint” [tiab] OR “resource constrained” [tiab] OR resource-constrained [tiab] OR developing [tiab] OR Africa [tiab] OR Asia [tiab] OR “South America” [tiab] OR “Latin America” [tiab] OR Latin-America [tiab] OR Afghanistan [tiab] OR Angola [tiab] OR Bangladesh [tiab] OR Benin [tiab] OR Bhutan [tiab] OR Bolivia [tiab] OR Burkina Faso [tiab] OR Burundi [tiab] OR Cabo Verde [tiab] OR Cape Verde [tiab] OR Cambodia [tiab] OR Cameroon [tiab] OR “Central African” [tiab] OR Chad [tiab] OR Comoros [tiab] OR “Congo, Dem. Rep.” [tiab] OR “Congo, Rep.” [tiab] OR Congo [tiab] OR “Côte d'Ivoire” [tiab] OR “Ivory Coast” [tiab] OR Djibouti [tiab] OR Egypt [tiab] OR “El Salvador” [tiab] OR Eritrea [tiab] OR Ethiopia [tiab] OR Gambia [tiab] OR Georgia [tiab] OR Ghana [tiab] OR Guinea [tiab] OR Guinea-Bissau [tiab] OR Haiti [tiab] OR Honduras [tiab] OR India [tiab] OR Indonesia [tiab] OR Kenya [tiab] OR Kiribati [tiab] OR “Korea, Dem. People's Rep.” [tiab] OR “North Korea” [tiab] OR Kosovo [tiab] OR “Kyrgyz Republic” [tiab] OR Kyrgyzstan [tiab] OR Kyrgyz [tiab] OR “Lao PDR” [tiab] OR Laos [tiab] OR Lao [tiab] OR

Kwete X, *et al. BMJ Global Health* 2021; 6:e005607. doi: 10.1136/bmjgh-2021-005607

	Lesotho [tiab] OR Liberia [tiab] OR Madagascar [tiab] OR Malawi [tiab] OR Mali [tiab] OR Mauritania [tiab] OR Micronesia [tiab] OR Moldova [tiab] OR Mongolia [tiab] OR Morocco [tiab] OR Mozambique [tiab] OR Myanmar [tiab] OR Nepal [tiab] OR Nicaragua [tiab] OR Niger [tiab] OR Nigeria [tiab] OR Pakistan [tiab] OR "Papua New Guinea" [tiab] OR Philippines [tiab] OR Rwanda [tiab] OR "São Tomé and Principe" [tiab] OR Senegal [tiab] OR Sierra Leone [tiab] OR Solomon [tiab] OR Somalia [tiab] OR Sudan [tiab] OR "Sri Lanka" [tiab] OR Sudan [tiab] OR Swaziland [tiab] OR Syrian [tiab] OR Tajikistan [tiab] OR Tanzania [tiab] OR Timor-Leste [tiab] OR Togo [tiab] OR Tunisia [tiab] OR Uganda [tiab] OR Ukraine [tiab] OR Uzbekistan [tiab] OR Vanuatu [tiab] OR Vietnam [tiab] OR "Viet Nam" [tiab] OR "West Bank and Gaza" [tiab] OR Yemen [tiab] OR Zambia [tiab] OR Zimbabwe [tiab] OR Albania [tiab] OR Algeria [tiab] OR Samoa [tiab] OR Armenia [tiab] OR Azerbaijan [tiab] OR Belarus [tiab] OR Belize [tiab] OR "Bosnia and Herzegovina" [tiab] OR Botswana [tiab] OR Brazil [tiab] OR Bulgaria [tiab] OR Colombia [tiab] OR "Costa Rica" [tiab] OR Cuba [tiab] OR Dominica [tiab] OR "Dominican Republic" [tiab] OR Ecuador [tiab] OR "Equatorial Guinea" [tiab] OR Fiji [tiab] OR Gabon [tiab] OR Grenada [tiab] OR Guatemala [tiab] OR Guyana [tiab] OR Iran [tiab] OR Iraq [tiab] OR Jamaica [tiab] OR Jordan [tiab] OR Kazakhstan [tiab] OR Lebanon [tiab] OR Libya [tiab] OR Macedonia [tiab] OR Malaysia [tiab] OR Maldives [tiab] OR Marshall [tiab] OR Mauritius [tiab] OR Mexico [tiab] OR Montenegro [tiab] OR Namibia [tiab] OR Nauru [tiab] OR Paraguay [tiab] OR Peru [tiab] OR Romania [tiab] OR "Russian Federation" [tiab] OR Russia [tiab] OR Samoa [tiab] OR Serbia [tiab] OR "South Africa" [tiab] OR "St. Lucia" [tiab] OR "St. Vincent and the Grenadines" [tiab] OR Suriname [tiab] OR Thailand [tiab] OR Tonga [tiab] OR Turkey [tiab] OR Turkmenistan [tiab] OR Tuvalu [tiab] OR Venezuela [tiab]))) AND "name of each institution"
Embase/Web of Science*	((global health OR global governance OR global diplomacy) OR ((Health OR Maternal OR child OR children OR Malaria OR Infectious OR disease surveillance OR Health policy OR health system OR health systems OR chronic illnesses OR non-communicable OR NCD OR Environmental health OR Climate change OR International development OR aid) AND (Low income OR low-income OR middle-income OR middle income OR resource constraint OR resource constrained OR resource-constrained OR developing OR Africa OR Asia OR South America OR Latin America OR Latin-America OR Afghanistan OR Angola OR Bangladesh OR Benin OR Bhutan OR Bolivia OR Burkina Faso OR Burundi OR Cabo Verde OR Cape Verde OR Cambodia OR Cameroon OR Central African OR Chad OR Comoros OR Congo OR Cote dIvoire OR Ivory Coast OR Djibouti OR Egypt OR El Salvador OR Eritrea OR Ethiopia OR Gambia OR Georgia OR Ghana OR Guinea OR Guinea-Bissau OR Haiti OR Honduras OR India OR Indonesia OR Kenya OR Kiribati OR Korea OR Kosovo OR Kyrgyz OR Kyrgyzstan OR Kyrgyz OR Lao OR Laos OR Lesotho OR Liberia OR Madagascar OR Malawi OR Mali OR Mauritania OR Micronesia OR Micronesia OR Moldova OR Mongolia OR Morocco OR Mozambique OR Myanmar OR Nepal OR Nicaragua OR Niger OR Nigeria OR Pakistan OR Papua

Kwete X, *et al. BMJ Global Health* 2021; 6:e005607. doi: 10.1136/bmjgh-2021-005607

	New Guinea OR Philippines OR Rwanda OR Sao Tome and Principe OR Senegal OR Sierra Leone OR Solomon OR Solomon OR Somalia OR Sudan OR Sri Lanka OR Sudan OR Swaziland OR Syrian OR Tajikistan OR Tanzania OR Timor-Leste OR Togo OR Tunisia OR Uganda OR Ukraine OR Uzbekistan OR Vanuatu OR Vietnam OR Viet Nam OR West Bank and Gaza OR Yemen OR Zambia OR Zimbabwe OR Albania OR Algeria OR Samoa OR Armenia OR Azerbaijan OR Belarus OR Belize OR Bosnia and Herzegovina OR Botswana OR Brazil OR Bulgaria OR Colombia OR Costa Rica OR Cuba OR Dominica OR Dominican OR Ecuador OR Equatorial Guinea OR Fiji OR Gabon OR Grenada OR Guatemala OR Guyana OR Iran OR Iraq OR Jamaica OR Jordan OR Kazakhstan OR Lebanon OR Libya OR Macedonia OR Macedonia OR Malaysia OR Maldives OR Marshall OR Mauritius OR Mexico OR Montenegro OR Namibia OR Nauru OR Paraguay OR Peru OR Romania OR Russian OR Russia OR Samoa OR Serbia OR Lucia OR (Vincent AND Grenadines) OR South Africa OR Suriname OR Thailand OR Tonga OR Turkey OR Turkmenistan OR Tuvalu OR Venezuela))) AND name of each institution
万方	(全球治理 + 全球外交 + 全球健康 + 全球卫生 + ((健康 + 儿童 + 母亲 + 疟疾 + 传染病 + 疾病监测 + 健康政策 + 卫生系统 + 慢性疾病 + 慢性病 + 环境健康 + 气候变化 + 国际发展 + 援助) * （低收入 + 中等收入 + 资源约束 + 不发达 + 贫困 + 亚洲 + 非洲 + 拉丁美洲 + 南美洲 + 埃塞俄比亚 + 博茨瓦纳 + 坦桑尼亚 + 加纳 + 尼日利亚 + 南非 + 乌干达 ））） * 作者单位:(每个单位的名称)

*Note that the search terms were modified for embase and web of science because neither of them can recognize special characters such as "Côte d'Ivoire".

Appendix Table 3. Name of each institution used in the search:

Institution	Database*	Name of institution used in the search
China National Health Development Research Center	Pubmed	(China[ad] AND National[ad] AND Health[ad] AND Development[ad] AND Research [ad] AND Center [ad])
	Embase	[affiliation] (China AND National AND Health AND Development AND Research Center)
	WoS	AD= (China AND National AND Health AND Development AND Research AND Center)
	WanFang	国家卫生计生委卫生发展研究中心
Dalian Medical University	Pubmed	(Dalian[ad] AND Medical[ad] AND University[ad])
	Embase	[affiliation] (Dalian AND Medical AND University)
	WoS	AD= (Dalian AND Medical AND University)
	WanFang	大连医科大学
Duke Kunshan University	Pubmed	(Duke[ad] AND Kunshan[ad])
	Embase	[affiliation] (Duke AND Kunshan)
	WoS	AD= (Duke AND Kunshan)
	WanFang	昆山杜克大学 / 杜克昆山大学
Sun Yat-sen University	Pubmed	(Sun [ad] AND yat-sen [ad])
	Embase	[affiliation] (Sun AND Yat-sen AND University)

	WoS	AD= (Sun AND Yat-sen AND University)
	WanFang	中山大学
Tsinghua University	Pubmed	(Tsinghua [ad])
	Embase	[affiliation] (Tsinghua)
	WoS	AD= (Tsinghua AND University)
	WanFang	清华大学
Fudan University	Pubmed	(Fudan[ad])
	Embase	[affiliation] (Fudan)
	WoS	AD= (Fudan AND University)
	WanFang	复旦大学
Peking University	Pubmed	(Peking[ad] AND University [ad])
	Embase	[affiliation] (Peking AND University)
	WoS	AD= (Peking AND University)
	WanFang	北京大学
Wuhan University	Pubmed	(Wuhan[ad] AND University [ad])
	Embase	[affiliation] (Wuhan AND University)
	WoS	AD= (Wuhan AND University)
	WanFang	武汉大学
China CDC	Pubmed	(China[ad] AND Center[ad] AND Disease[ad] AND Control [ad])
	Embase	[affiliation] (China AND Center AND Disease AND Control)
	WoS	AD= (China AND Center AND Disease AND Control)
	WanFang	中国疾病预防控制中心 / 中国疾控中心
Xi'an Jiao Tong University	Pubmed	((Xi'an[ad] OR Xian [ad]) AND((Jiao[ad] AND tong[ad]) OR Jiaotong [ad]) AND University[ad])
	Embase	[affiliation] (Xian OR (Xi AND an)) AND (Jiaotong OR (Jiao AND Tong)) AND University)
	WoS	AD= (Xian OR (Xi AND an)) AND (Jiaotong OR (Jiao AND Tong)) AND University
	WanFang	西安交通大学

Appendix Table 4. Search results of the bibliometric analysis:

**Institution**	**Pubmed**	**Embase**	**Web of Science (ISI)**	**Wanfang**	**Total after duplicates removed**	**After title and abstract review**	**After full text review**
NHDRC	99	6	319	28	368	268	11
DMU	43	9	165	255	440	34	15
DKU	36	6	64	1	73	49	39
SYS	289	76	1397	1177	1859	132	94
THU	102	8	1215	997	2133	98	67
FU	319	58	1435	2311	3264	251	204
PKU	569	152	2564	2041	4823	522	266
WU	404	34	2817	835	3640	188	73
CDC	755	44	2909	736	3837	445	280
XJTU	92	34	696	336	1064	183	36

Kwete X, *et al. BMJ Global Health* 2021; 6:e005607. doi: 10.1136/bmjgh-2021-005607

Appendix Table 5. Interview guides used for the key formants' interviews:

Section	Questions
1	Name of institute; name of department; position at the institute and role
2	What's your understanding of global health research? How about China-Africa Health collaboration?<br>What is the goal of global health research for this institute? How about in low- and middle-income countries specifically? How about for Africa?<br>Are there any barriers to conducting global health research? If yes, what are they? How about for low- and middle-income countries specifically? How about for Africa?<br>What have you done to overcome those barriers? Please provide answer to each of the barriers listed above.<br>What support do you need in strengthening the global health research capacity of your institute? In what specific areas? How about for low- and middle-income countries specifically? How about for Africa?<br>What is your expectation of global health research capacity of your institute in the next 5 years? 10 years? What's the specific expectation in research capacity in low- and middle-income countries? What's the specific expectation in research capacity in Africa?
3	On his or her opinion regarding the research capacity of other global health institutes in China.<br>What is your expectation for the research capacity of Chinese global health institutes collectively in the next 5 to 10 years? How about for low- and middle-income countries specifically? How about for Africa?

Kwete X, *et al. BMJ Global Health* 2021; 6:e005607. doi: 10.1136/bmjgh-2021-005607

Appendix Table 6. Goals for global health research by institution:

Institution name	Health promotion	Research/knowledge generation	Human resource training	Policy support
Duke Kunshan University	"to improve the health and welfare of all human beings and to eliminate the health inequity."			
Fudan School of Nursing	"to carry out the global health research to promote more effective and sustainable public health"			
Department of Global Health, PKU	"to promote equity and to ensure and accessibility of health. to help each country find their solution based on their own local context, to ensure a basic level of health for all."	"to look for a brand-new solution and perspective through global collaboration."	"to train future talents. to train talents with global perspective and as academic leaders."	"to build partners, with Africa and South Asia."
National Institute for Parasitic Diseases, China CDC	"to share the results of our successful experience in combating tropical diseases with our African partners. to solve local health issues, using the technologies China already acquired."			"to promote the progress and level of China Africa health collaboration and global health collaboration."
National Health and Development Research Center				"to serve the policy and the policy-making process"
Wuhan University Global Health Research Center	"to care for global health, the development of all human society and people's well-being."		"exchange of experience and capacity building in developing countries."	
Xi'an Jiaotong University Global Health Institute	"to promote the public health in developing countries."	"to research the heated topics on health from a global perspective."	"to train more talents in this area through global health related projects."	"to support the Belt and Road Initiative."

*only partial results were listed based on their requests for confidentiality.

Kwete X, *et al. BMJ Global Health* 2021; 6:e005607. doi: 10.1136/bmjgh-2021-005607

<u>Appendix Table 7.</u> Common feature on the expectation of research capacity development for the next 5-10 years.

Theme	Quotes
Strong will to improve the overall research capacity	"We hope we will play an important role for promoting African countries and to rebuild health systems and to achieve the goal of SDGs."<br><br>"In the next five to ten years, I hope we could reach the global health research like that in developed countries."
Optimistic expectation for the overall research capacity and expectation to grow out of the current "infant phase"	"From next year or the year after, we will be able to publish a bunch of very impactful research. Our expectation is to have concrete academic achievements at the 5-year anniversary."<br><br>"We look forward to applying for another global health project independently in the next 5 years."<br><br>"We expect to have strong capacity in talent training and education, to increase both the quantity and quality of talents in global health, in order to meet the growing demands of global health."<br><br>"We expect … to become a leading institute domestically and internationally, and become very productive in global health research, with at least 20-30 publications every year."
Emphasis on a growing impact to and closer ties with other low- and middle-income countries, especially Africa	"We want to undertake participate in cooperative research, consulting and training in Africa countries."<br><br>"We will continue cooperating with Ethiopian's train-the-trainer project; this is a project for the next 10 years."<br><br>"We also hope we could efficiently help Africa and other low and middle-income countries to detect problems and propose systematic solutions that fit into local context."<br><br>"My vision for the institute is to become a pioneer in China's health collaboration with Africa, and to have some flagship programs, with certain impact in Africa."<br><br>"In 5-10 years, I think we can at least have a solid foot in Africa."<br><br>"I also hope we would have a few projects that make real contribution to the life of the people in Africa, to let the China experience be rooted in the Africa continent."<br><br>"Africa hasn't been our main field in the past, but we will work to develop better ties with Africa in the next 5-10 years."<br><br>"We hope to have some collaboration projects in health systems, in addition to the vertical disease specific public health programs and the traditional medical team program, to really help improve their national health systems and health policy

	making process, supporting their health care reform. If we can share some good experience with even just a few countries but making real impact in improving their national health policy making process and the health system, it would be very good and will have long lasting impact."
Expansion on existing or establishment of potential networks/partners	"…we hope to have more collaborators in Africa, and together we can explore a global health mode with Chinese characteristics."<br><br>"Now we already have established the China Africa Schistosomiasis Network with 10 members in Africa, and the China Africa Malaria Network with 5 members. We hope each of numbers could reach 20-30 within the 5-10 years."<br><br>"In terms of projects, we now have one site for schistosomiasis, and one site for malaria. We hope to have 5 sites within 5 years and 10 sites within 10 years, with regular collaborators pool, to form a concrete and substantiate partner structure with field sites, networks and strong collaborators."<br><br>"We already have some international cooperation projects; but we hope to expand on that and make them really successful and provide our input to the Belt and Road Initiative."

Kwete X, *et al. BMJ Global Health* 2021; 6:e005607. doi: 10.1136/bmjgh-2021-005607
